# PLOD3 contributes to HER-2 therapy resistance in gastric cancer through FoxO3/Survivin pathway

**DOI:** 10.1038/s41420-022-01103-4

**Published:** 2022-07-14

**Authors:** Yueda Chen, Botian Ye, Chunyan Wang, Yanyan Nie, Jing Qin, Zhenbin Shen

**Affiliations:** 1 Department of General Surgery, Zhongshan Hospital (Xiamen), Fudan University, Xiamen, Fujian 361015 China; 2 Department of General Surgery, Gastric Cancer Institute, Zhongshan Hospital, Fudan University, Shanghai, 200032 China; 3 Department of Obstetrics and Gynecology, Tenth People’s Hospital of Tongji University, Shanghai, 200072 China; 4 Shanghai Lab, Animal Research Center, Shanghai, 201203 China

**Keywords:** Gastric cancer, Cancer therapeutic resistance, Cancer metabolism

## Abstract

Human epidermal growth factor receptor 2 (HER-2), a famous therapeutic target for breast cancer, is also associated with an increased risk of recurrence and poor outcomes of other malignancies, including gastric cancer. Yet the mechanism of HER-2 therapy resistance remains controversial due to the heterogeneity of gastric adenocarcinoma. We know, Procollagen-Lysine,2-Oxoglutarate 5-Dioxygenase 3 (PLOD3), a key gene coding enzymes that catalyze the lysyl hydroxylation of extracellular matrix collagen, plays an important contributor to HER-2 targeting agent Trastuzumab resistance in gastric cancer. Herein, we analyzed clinical samples of gastric cancer patients and gastric cancer cell lines and identified PLOD3, unveiled that depletion of PLOD3 leads to decreased cell proliferation, tumor growth and Trastuzumab sensitivity in these Trastuzumab resistant GC cell lines. Clinically, increased PLOD3 expression correlates with decreased Trastuzumab therapy responsiveness in GC patients. Mechanistically, we show that PLOD3 represses tumor suppressor FoxO3 expression, therefore upregulating Survivin protein expression that contributes to Trastuzumab resistance in GC. Therefore, our study identifies a new signaling axis PLOD3-FoxO3- Survivin pathway that may be therapeutically targeted in HER-2 positive gastric cancer.

## Introduction

In 2020, over one million new cases of stomach cancer are expected worldwide, and over 769,000 deaths are anticipated, ranking it fifth for incidence and fourth for mortality [1]. There are many patients who present with advanced, metastatic or inoperable disease that requires palliative care. Further, it is generally more prevalent in Eastern Asia than in Europe or North America [2, 3].

It is known that Human epidermal growth factor receptor 2 (HER-2), increases the risk of recurrence and adverse outcomes of certain cancers, including gastric cancer [4]. A recent study found that HER-2 plays a key role in cancer initiation and progression, and that dysregulation of HER-2 affects gastric cancer prognosis independently [5]. HER-2 is present in approximately 20% of advanced gastric cancers [6]. The phase III ToGA trial showed that chemotherapy plus Trastuzumab, a HER-2- targeted monoclonal antibody, improved overall survival (13.8 months vs. 11.1 months) and progression-free survival (6.7 months vs. 5.5 months) in patients with these cancers versus chemotherapy alone [7]. Although Trastuzumab’s response rate varied from 16-29%, it was relatively low [8]. As a consequence, identifying the mechanism behind Trastuzumab resistance in gastric cancer is essential to developing new therapies.

Based on previous studies, we aim to characterize the role of PLOD3, a key enzyme that catalyzes the lysyl hydroxylation of extracellular matrix collagen, in gastric cancer. In addition, we sought to examine the role of PLOD3 on mediating Trastuzumab resistance in gastric cancer and its underlying mechanism by investigating the involvement of FoxO3/Survivin pathway.

## Results

### Identification of potential important genes responsible for Trastuzumab resistance in gastric cancer

**Fig. 1:**
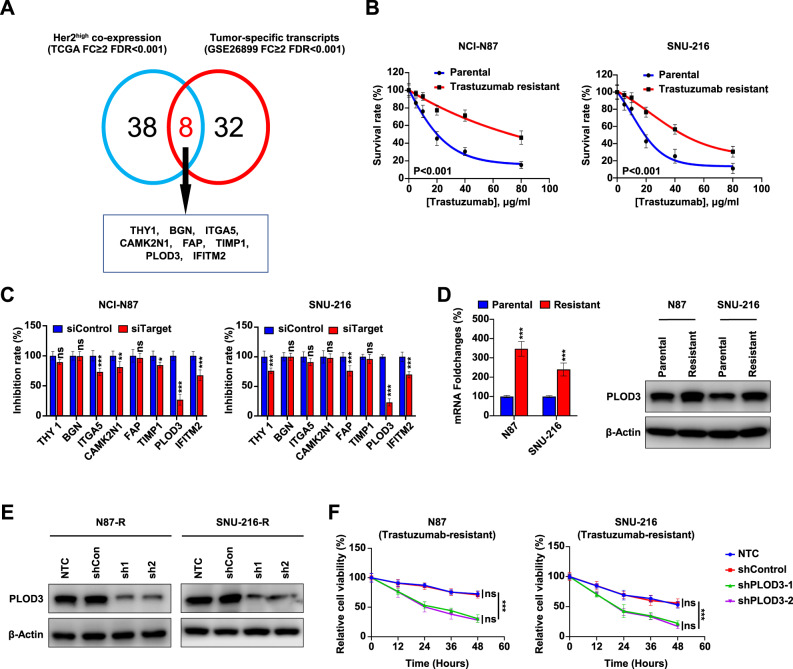
Identification of PLOD3 as a novel regulator responsible for Trastuzumab resistance in gastric cancer. **A** Analysis of genes responsible for Trastuzumab resistance in gastric cancer from TCGA database and RNA-Seq database(GSE26899). **B** The trastuzumab-resistant GC cell lines were generated by treating HER-2 positive parent cell lines N87 and SNU-216 with trastuzumab, and the resistance potentials were verified by MTT assays. **C** The siRNA depletion of indicated targets were conducted in NCI-N87 and SNU-216 cells followed by MTT assays to determine the inhibition efficiency. Silencing PLOD3 significantly decreased cell proliferation when compared to other genes when treated with Trastuzumab. **D** Expression of PLOD3 in Tr cells and parental cells were determined by quantitative PCR and western blot. **E** Established Tr cell lines with PLOD3 depletion by two independent shRNAs (sh1, sh2) and validated by western blot. **F** The proliferation in both N87 and SUN-216 Tr cells were confirmed by MTT.

To identify the potential regulator in promoting Trastuzumab resistance in gastric cancer (GC), we employed a two-step screening approach. First, we hypothesized that there may exist genes that co-express with HER-2 that may directly or indirectly contribute to Trastuzumab resistance. To this end, we investigated to identify the strongly correlated genes with HER-2 expression in GC cancer patients from TCGA database, which might exert vital regulatory role in mediating response to Trastuzumab. Second, we also screened differential expressed transcripts in GC compared to other cancers, termed GC-specific transcripts from a previous RNA-Seq database from GC patients (GSE26899). Next, we overlapped these two lists and found 8 overlapping genes including THY1, BGN, ITGA5, CAMK2N1, FAP, TIMP1, IFITM2, and PLOD3. Therefore, we identified a short list of genes that may co-express with HER-2 and their expression was upregulated in GC (Fig. 1A).

### PLOD3 contributes to Trastuzumab resistance in GC in vitro

To further identify the key regulator that promoting Trastuzumab resistance in GC, we generated two Trastuzumab‐resistant GC cell lines by subjecting HER-2 positive parental cell lines N87 and SNU-216 to long-term Trastuzumab treatment, naming them N87/Tr and SNU-216/Tr respectively. These Trastuzumab resistant GC cells displayed significantly elevated IC50 values for Trastuzumab compared to their parental counterparts according to MTT assay results (Fig. 1B). To identify which genes described above may contribute to resistance, we implemented siRNA strategy to deplete THY1, BGN, ITGA5, CAMK2N1, FAP, TIMP1, IFITM2 or PLOD3 expression respectively followed by the cell proliferation assay in these Trastuzumab resistant cell lines. Our results showed that PLOD3 depletion by the siRNA significantly decreased cell proliferation, in comparison to other genes when treated with Trastuzumab (Fig. 1C). Since PLOD3 depletion gave rise to the most robust and consistent cell proliferation defect among these two cell lines, we decided to focus on characterizing the role of PLOD3 in this process. Moreover, PLOD3 expression displayed also significantly increased expression in Tr cells compared to parental cells (Fig. 1D). Importantly, we established Tr cell lines with PLOD3 depletion by two independent shRNAs (sh1, sh2). There are significantly decreased PLOD3 abundance in shPLOD3 N87 knockdown groups than the control groups (Fig. 1E). PLOD3 depletion by these shRNAs led to significantly decreased cell proliferation in both N87 and SNU-216 Tr cells (Fig. 1F). Therefore, our findings suggest that PLOD3 upregulation is associated with, and may contribute to, Trastuzumab resistance in HER-2 positive gastric cancer.

### In vivo verification of PLOD3 as the key regulator for Trastuzumab resistance in GC

**Fig. 2:**
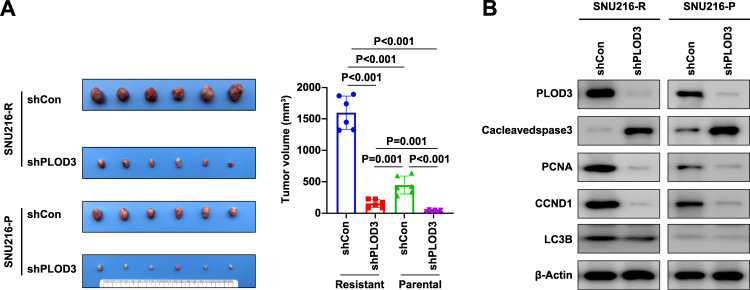
PLOD3 was the key regulator for Trastuzumab resistance in gastric cancer in vivo. **A** In vivo evaluation of the effect of PLOD3 on tumor proliferation. **B** Examined cell proliferation and apoptosis of indicated tumor tissues derived from in vivo models by western blot.

Motivated by our in vitro findings, we aimed to determine the effect of PLOD3 on mediating Trastuzumab resistant GC tumor growth in vivo. We implanted both parental and Trastuzumab resistant cells with or without PLOD3 knockdown into the mice followed by Trastuzumab treatment for 5 weeks. We noticed that Trastuzumab resistant cells displayed significantly enhanced tumor growth compared to parental cells, validating its resistant nature in vivo. Second, PLOD3 depletion led to significantly decreased tumor growth in the mice (Fig. 2A), validating the critical role of PLOD3 in mediating Trastuzumab resistance. In addition, we also extracted cell lysates from these tumors and confirmed PLOD3 depletion in these PLOD3 shRNA infected cells, suggesting that the decreased tumor growth may derive from PLOD3 depletion. Furthermore, we also examined some of cell proliferation and apoptosis markers. PLOD3 depletion in vivo displayed decreased cell proliferation markers including CCND1 and PCNA whereas apoptosis marker cleaved caspase 3 level was increased (Fig. 2B). Interestingly, LC3B, a symbol of autophagy procedure, was also increased in Trastuzumab-resistant GC cells, but showed only slight decreased when PLOD3 was knocked down, indicating PLOD3 might only partially regulated Trastuzumab resistance via modulating autophagy. In summary, our results suggest that PLOD3 is important for contributing to Trastuzumab resistance in GC.

### PLOD3 expression states associated with Trastuzumab treatment efficiency in HER-2-positve GC patients

**Fig. 3:**
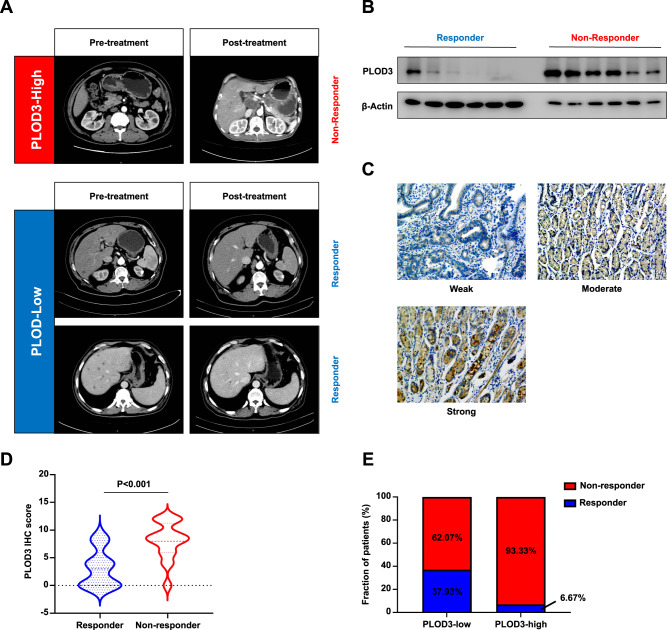
The expression of PLOD3 is associated with Trastuzumab effectiveness in HER-2 positive GC patients. **A** Representative images of Patients with distinct PLOD3 expression as well as the response to Trastuzumab were shown. **B** The protein levels of PLOD3 in responder and nonresponder GC patients receiving Trastuzumab were assessed by western blot. **C** Representative images of IHC staining for PLOD3 in clinical GC samples. **D** IHC score of PLOD3 in GC patients receiving Trastuzumab. **E** Response rate of gastric cancer patients with different PLOD3 expression status to trastuzumab therapy.

To further evaluate the predictive value of PLOD3 expression in GC patients undergoing Trastuzumab treatment, 74 patients with advanced HER-2 positive gastric cancer were investigated retrospectively. Among all these patients, 14 individuals showed response to Trastuzumab treatment. Representative images were shown as (Fig. 3A). We first conducted immunoblot assays to examine the expression pattern of PLOD3 between responders and nonresponders. Very interestingly, responders displayed significantly lower PLOD3 protein levels compared to those non-responders (Fig. 3B). We also performed orthogonal approaches by staining these tumors for PLOD3 by IHC (Fig. 3C). In accordance with our western blot analysis, we observed that non-responders had significantly higher PLOD3 expression than responders did (*P* < 0.001, Fig. 3D). Further investigation showed a drastic higher percentage of responder in PLOD3-low subgroup (low 37.93% vs high 6.67%, Fig. 3E). Taken together, above data indicated PLOD3 expression level was a promising predictor for evaluating response to Trastuzumab treatment in GC patients.

### PLOD3 promotes HER-2-positive GC progression in vitro

**Fig. 4:**
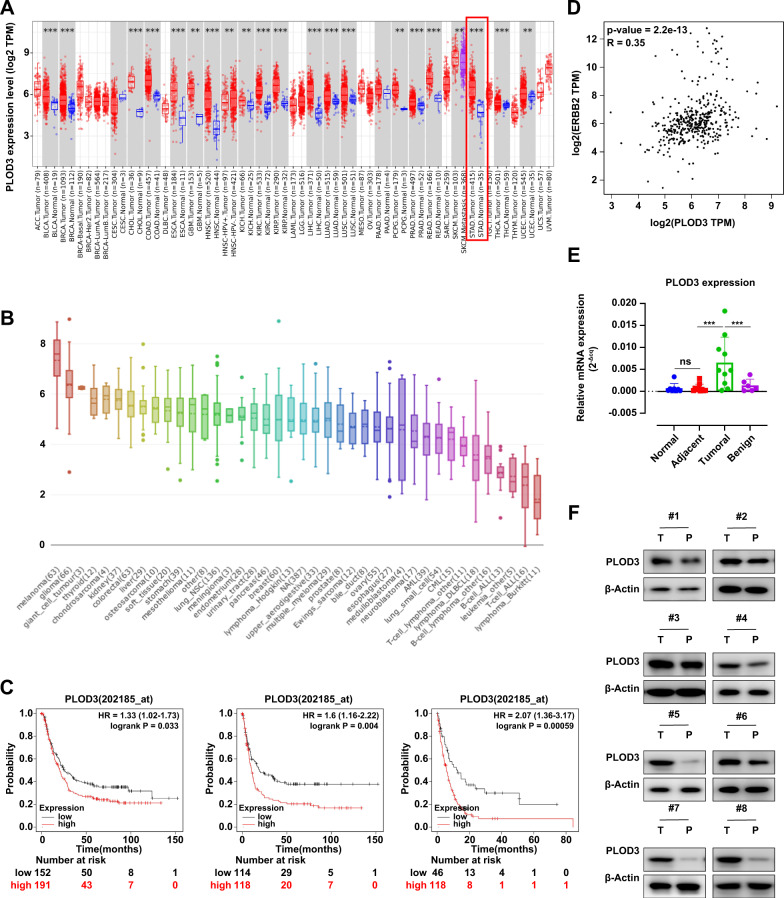
PLOD3 was essential for GC progression. **A** Comparisons of PLOD3 mRNA expression levels between normal and cancerous tissues according to TCGA database. **B** PLOD3 displayed high expression pattern in GC cell lines according to CCLE database. **C** Kaplan-Meier analysis of overall survival, first progression and postprogression survival of patients according to PLOD3 expression status (high or low). **D** The association between PLOD3 expression and HER-2 expression according to STAD dataset of TCGA database. **E**, **F** PLOD3 was highly expressed in GC patients (T) compared to paired normal tissues (P) by conducting RT-PCR and immune-blotting assays.

Since the biological function of PLOD3 remained elusive in GC, we also explored whether PLOD3 was essential for GC progression even without Trastuzumab treatment. First, we found PLOD3 expression showed universally high expression pattern in variety types of solid tumors including GC according to TCGA database (Fig. 4A). Also, PLOD3 displayed high expression pattern in GC cell lines according to CCLE database (Fig. 4B). In addition, PLOD3 expression was closely associated with HER-2 expression in GC (Fig. 4D, *R* = 0.35). Importantly, patients with high PLOD3 expression showed significantly shorter OS (*P* = 0.033), FP (*P* = 0.004), and PPS (*P* < 0.001) in HER-2-positive subgroup in GC patients according to Kaplan-Meier Plot database (Fig. 4C). Moreover, using our own cohort data, we found PLOD3 was highly expressed in GC patients (T) compared to paired normal tissues (P) by conducting RT-PCR and immune-blotting assays (Fig. 4E, F).

**Fig. 5:**
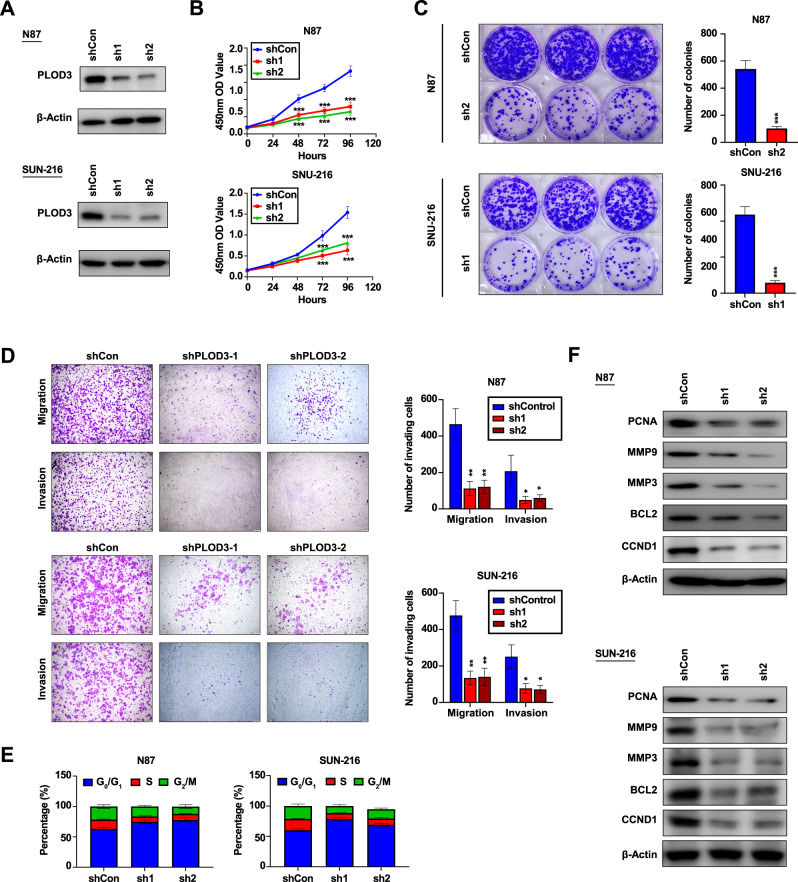
The biological function of PLOD3 in vitro. **A** PLOD3 expression was knocked down by lentivirus in N87 and SNU-216 cell lines, and knockdown efficiencies were validated by immune-blotting experiments. **B**, **C** Inhibitory effects of PLOD3 downregulation on the proliferation of PLOD3-high GC cells were validated by colony formation assays and MTT assays. **D** Inhibitory effects of PLOD3 downregulation on the migration and invasion capacities of PLOD3-high GC cells were validated by transwell assays. **E** The cell cycle variation after PLOD3 knockdown in GC cells line according to flow cytometry assays. **F** The expression levels of invasion and proliferation-related markers were assessed by western blot.

Next, we focus on the biological function of PLOD3 in HER-2-positive GC cells. PLOD3 expression was knocked down by lentivirus in N87 and SNU-216 cell lines, and knockdown efficiencies were validated by immune-blotting experiments (Fig. 5A). MTT and colony formation assays showed that down-regulation of PLOD3 expression resulted in impairment of proliferation in HER-2-positive GC cells (Fig. 5B, C). Transwell assays revealed PLOD3 knockdown significantly hindered migration as well as invasion capacities in both two HER-2-positive GC cell lines (Fig. 5D). Moreover, PLOD3 knockdown resulted in G0/G1 arrest according to flow cytometry assays (Fig. 5E). Concordantly, expression levels of several invasion or proliferation-related markers such as PCNA, MMP9, and CCND1 were impressively inhibited by PLOD3 knockdown (Fig. 5F). Together, our results above suggest that PLOD3 is critical for HER-2-postive GC progression.

### PLOD3 exerts its function mainly by inhibiting FoxO3 expression

**Fig. 6:**
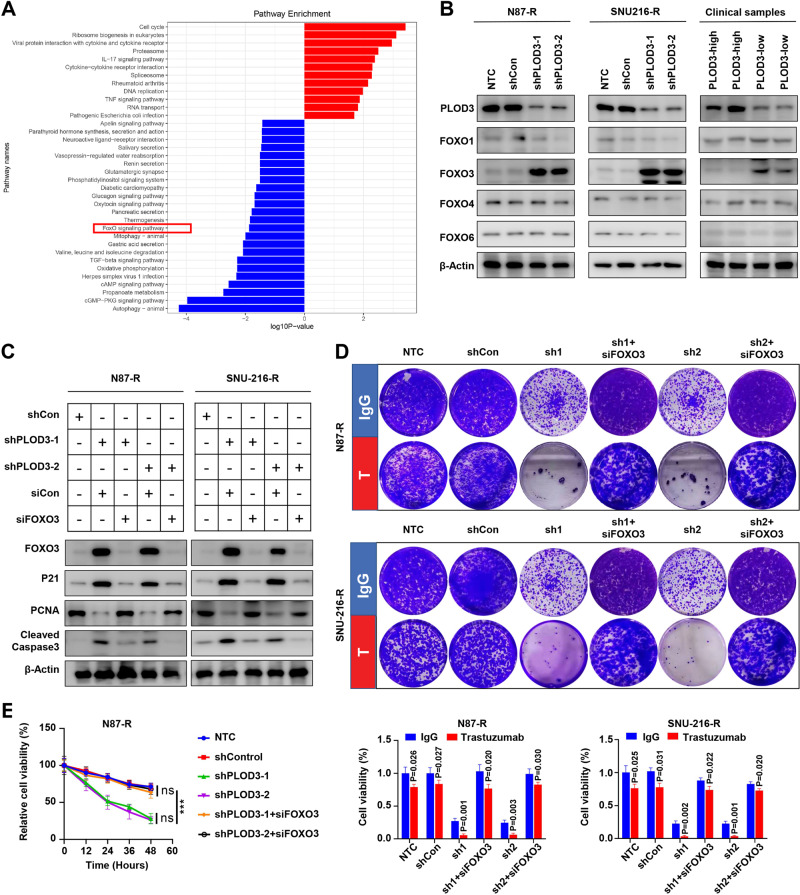

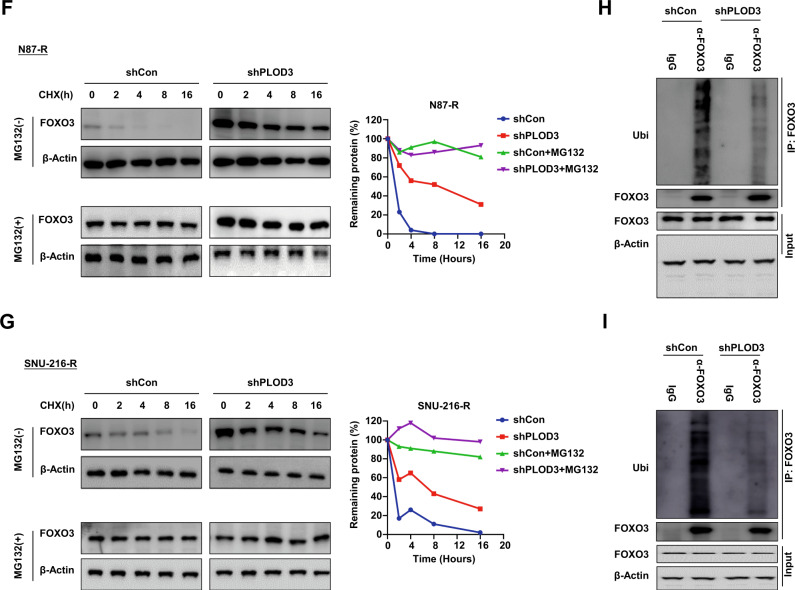
PLOD3 decreased protein stability of FOXO3 to promote Trastuzumab resistance in GC. **A** KEGG analysis was performed to investigate significantly altered signaling pathways between PLOD3-high and PLOD3-low subgroup in GC according to TCGA database. **B** FOXO3 showed dramatically increased expression after PLOD3-KD according to western blot. **C** FOXO3 silencing could effectively rescue the inhibitory effects of PLOD3 knockdown on p21 and cleaved caspase3 expressions under Trastuzumab treatment. **D** Silencing FOXO3 could restore Trastuzumab resistance in PLOD3 knockdown GC cells according to colony formation assays. **E** FOXO3 depletion reversed the cell proliferation inhibition of PLOD3 knockdown in GC cells under Trastuzumab treatment according to MTT assays. **F**, **G** The protein stability of FOXO3 after PLOD3 knockdown in N87R and SNU-216R cells were conducted by CHX pulse-chase experiments. **H**, **I** PLOD3 knockdown affected FOXO3’s ubiquitination level according to ubiquitination assays.

To identify key mediators involved in PLOD3-induced HER-2 resistance, we performed pathway enrichment analysis to investigate significantly altered signaling pathways between PLOD3-high and PLOD3-low subgroup in GC. Interestingly, we observed FoxO signaling pathway was one of the most significantly downregulated pathways in PLOD3-low subgroup in gastric cancer (Fig. 6A). Considering FoxO signaling exerted extensive inhibitory effects on tumor cell proliferation and progression [9], we thus, focus on this signaling pathway. Previous study reported that FoxO1 and FoxO3 were two major participants involved in regulating tumor cell survival under Trastuzumab treatment [10], therefore, we evaluated the expression states of FoxO1, FoxO3, FoxO4 and FoxO6. Surprisingly, we found that only FoxO3 showed dramatically increased expression due to PLOD3 knockdown, whereas other three FoxO members exerted no overt change (Fig. 6B). Moreover, clinical samples also revealed that high-PLOD3 GC tumors tissues correlated with low FoxO3 expression. To validate the role of FoxO3, we silenced FoxO3 expression via siRNAs in PLOD3-KD Trastuzumab-resistant GC cells, and measured p21, PCNA, and cleaved Caspase3 expression via immunoblot assays. Results showed that silencing FoxO3 could effectively rescue the inhibitory effects of PLOD3 knockdown on PCNA expression whereas repressing the levels of p21 and cleaved Caspase3 (Fig. 6C). Further colony-formation experiments also indicated that silencing FoxO3 could successfully restore Trastuzumab resistance in PLOD3 knockdown GC cells, evidenced by significantly increased colony numbers (Fig. 6D). Similarly, MTT assays demonstrated that FoxO3 depletion in PLOD3 knockdown GC cells rendered these cells to proliferate under Trastuzumab treatments (Fig. 6E). Together, these data verified that PLOD3 promotes Trastuzumab resistance mainly by inhibiting the expression of FoxO3.

### PLOD3 inhibits FoxO3 through ubiquitination degradation pathway

Next, we further investigated the underlying mechanism by which PLOD3 represses FoxO3. We observed the mRNA expression of FoxO3 after PLOD3 knockdown, however, no significant alteration was observed. Thus, we conducted CHX pulse-chase experiments to investigate the protein stability of FoxO3. Our results showed that PLOD3 knockdown dramatically increased the protein stability of FOXO in both Trastuzumab-resistant GC cells (Fig. 6F, G). Under most circumstances, intracellular protein stability is mainly regulated by the proteasome or lysosomal pathway. Hence, we conducted ubiquitination assays to evaluate the influence of PLOD3 knockdown on the ubiquitination level of FoxO3. We found that PLOD3 knockdown could effectively decreased the ubiquitination level of FoxO3 in both two Trastuzumab resistant GC cells (Fig. 6H, I). Together, our data indicated PLOD3 inhibits FoxO3 expression through inducing its ubiquitination and promotes FoxO3 degradation by the proteasome pathway.

### FoxO3-Survivin pathway is crucial for PLOD3-induced HER-2 resistance

**Fig. 7:**
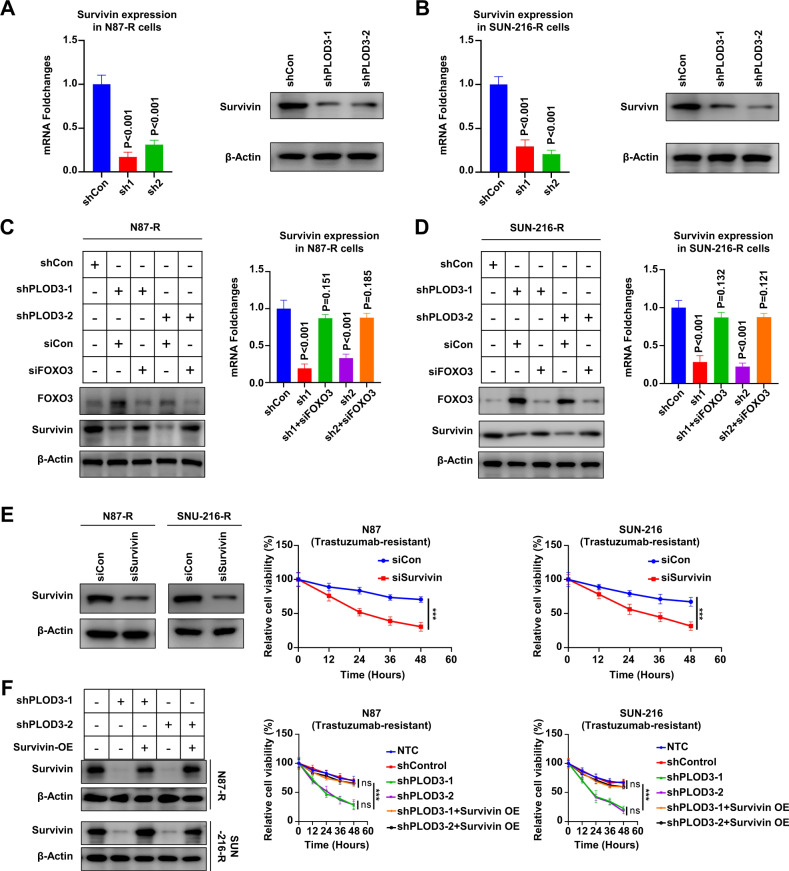
The FOXO3-Survivin pathway plays a crucial role in PLOD3-induced resistance to HER-2. **A**, **B** The mRNA and protein levels of Survivin after PLOD3 knockdown in Trastuzumab-resistant GC cell lines were validate by RT-PCR and western blot. **C**, **D** RT-PCR and immune blotting analysis showed that silencing FOXO3 in PLOD3-knockdown cells could restore Survivin expression. **E** MTT assays indicated suppression of Survivin expression in Trastuzumab-resistant GC cells could resulted in sensitization towards Trastuzumab. **F** Trastuzumab-resistant potential could be restored by overexpressing Survivin in PLOD3-knockdown GC cells according to MTT assays.

Previous study reported Survivin was the key downstream target of FoxO3 in Trastuzumab-resistant cells. Therefore, we speculated PLOD3 might also promote Trastuzumab resistance via FoxO3/Survivin axis. To validate this, we explored the expression of Survivin after PLOD3 knockdown. Our results showed that PLOD3 knockdown by two different shRNAs led to decreased Sruvivin expression on both mRNA and protein levels in multiple GC Trastuzumab resistant cell lines (Fig. 7A, B). Meanwhile, silencing FoxO3 expression in PLOD3 knockdown cells could restore Survivin expression, illustrated both by RT-PCR and immune-blotting assays (Fig. 7C, D). Importantly, silencing Survivin expression in Trastuzumab resistant GC cells could induce GC cells sensitized to Trastuzumab according to MTT assays (Fig. 7E). On the contrary, forced expression of Survivin in PLOD3 knockdown GC cells could restore the Trastuzumab-resistance potentials (Fig. 7F). Taken together, above results indicated that PLOD3 exerted its drug-resistance promoting capacity via inducing FoxO3 protein degradation, which resulted in abundant Survivin expression, and led to Trastuzumab resistance formation in GC cells.

## Discussion

In our study, we show that PLOD3 is a key mediator for HER-2 resistance through downregulating FoxO3 therefore promoting survivin pathway in gastric cancer. Depletion of PLOD3 leads to increased Trastuzumab sensitivity by using in vitro and in vivo GC models. In addition, patients in PLOD3-high group also show higher possibility of developing Trastuzumab resistance in GC cancer.

Currently several randomized clinic trials have been focusing on the possible mechanism of HER-2 resistance. Anti-HER-2 antibody trastuzumab deruxtecan has significantly improved the response rate (51% vs. 14%) and overall survival (12.5 vs. 8.4 months) of HER-2 positive advanced gastric cancer patients compared with chemotherapyr [11], indicating that trastuzumab deruxtecan may be less affected by high HER-2 expression due to its high drug-to-antibody ratio and membrane permeability. Compared to trastuzamab, pertuzumab targets HER-2 receptor protein epitopes differently and has shown considerable improvement in survival rates.Combined with trastuzumab chemotherapy, pertuzumab has shown to significantly improve survival outcomes for HER2-positive breast cancer,has demonstrated potential therapeutic benefit in colorectal cancers with HER-2 amplification, but failed in HER-2 positive gastric cancers [12, 13].

Procollagen-lysine,2-oxoglutarate 5-dioxygenase (PLOD) catalyzes the lysyl hydroxylation of extracellular matrix collagen [14]. PLOD3 is distinguished from PLOD1 and PLOD2 in terms of its enzymatic activity for GT and GGT [15]. Increased collagen deposition and cross-linking may promote cancer development and progression by promoting the invasion, migration, and proliferation of cancer cells [16], An animal model of spontaneous liver cancer has been reported to suppress liver tumor incidence through knockdown of PLOD3 [17]. To our knowledge, the characterization of PLOD3 in gastric cancer remains unclear to date. Not only do our results discover upregulated expression of POLD3 in GC cell lines, GC xenograft models and clinical tumor tissue, but also link PLOD3 expression with poor survival outcomes in GC patients. We noticed that knockdown of PLOD3 significantly suppressed cell viability, reduced migration and invasiveness of tumor cells. These results could enlighten us with further understanding ofphysiological and tumorigenic function of PLOD3.

As a member of the forkhead box O (FOX) superfamily, FoxO3 plays an important role in regulating a range of biological processes such as development, cell signaling and tumorigenesis in the process of cell metabolism [18]. It should be noted that FoxO family members are phosphorylated by PI3K/AKT, the pathway associated with HER-2 resistance in many types of cancer^14^. Beyond AKT and PKA phosphorylation, FoxO3 may also be phosphorylated by other serin/threonine kinases. For example, MST1 phosphorylates S207 [19], ERK-1/2 phosphorylates S295/345/426, AMPK phosphorylates S413/588/626 [20], and IKKb phosphorylates S644 [21, 22]. Invasion and metastasis are suppressed by FoxO3 through its positive regulation of E-cadherin and negative regulation of Twist1 [23]. FoxO3 could also be activated by other transcription factors, including FOXK2 [24], glucocorticoid receptor (GR) [25] and AP1 [26] could also increase the transcriptional activity of FoxO3. Before our research, few documents have reported the relationship between PLOD3 and FoxO3 in tumorigenesis. Therefore, our research indicated a novel potential target for treating and avoiding resistance in HER-2-positive gastric cancer.

Our research had a few limitations. For example, our study is based on cell line studies with a small cohort of patient samples in GC. The definition of HER-2-positive gastric cancer is still universally controversial with regarding to methods of detection and specific cut-off defining positivity. Also, the underlying mechanism by which PLOD3 regulates FoxO3 protein stability remains unclear. Further investigations need to be carried out to embark on these areas.

In conclusion, we show that PLOD3 is a key mediator for HER-2 resistance through downregulating the expression of FoxO3 therefore upregulating Survivin pathway in gastric cancer. Our research uncovers a novel potential signaling axis for treating and reversing resistance in HER-2-positive gastric cancer.

## Materials and methods

### Patient specimens

Gastric cancer (GC) samples were taken from 74 patients who had been treated with radical resections at Zhongshan Hospital of Fudan University, China, between August 1, 2011 and June 30, 2014. Informed consents were signed by the patients or their relatives and was approved and documented (No. 2019-KY-93) by the ethics committee of Zhongshan Hospital of Fudan University. From the same patient, we obtained cancer and normal tissue samples. Patients who were pathologically diagnosed gastric adenocarcinoma within stage T 1–4N0–M0 were chosen for these study. Within these patients who has received chemoradiotherapy before operation, had targeted drug therapy like Tratuzumab for Her 2 positive cases, or those died of diseases other than GC, e.g., cardiac and cerebrovascular accidents during the follow-up period were excluded from the study. All the patients should have tissue sections and clinical data were kept intact and during the treatment and follow-up period. The survival time of the patients were counted from the date of surgery to the date of death. The end of the follow-up was on June 30, 2019. Gastric cancer was classified according to the WHO’s classification of digestive tumors, 2019 [27], and the clinical staging was based on the TNM staging of GC in the 8th edition formulated by the American cancer society (AJCC) and the international union against cancer (UICC) in 2017 [28].

### Cell lines

We purchased N87 and SNU-216 from the cell bank built by Chinese Academy of Sciences (Shanghai, China). Both two cell lines were maintained in medium DMEM (Gibco) with 10% FBS (Gibco). Incase tie cell would be contaminated, penicillin plus streptomycin were added with a concentration of 100 U/ml. Throughout the study period, cells were cultured at 37 °C under 5% CO_2_ and regularly tested by Mycoplasma Detection Kit (Roche Diagnostics).

### RNA extraction, cDNA generation, and quantification by reverse transcription-polymerase chain reaction (qRT-PCR)

We extracted RNA from cells and frozen tumor tissue with RNeasy mini kit (Qiagen). As soon as possible, total mRNA concentration was measured, and reverse transcription was completed by QuantiTect reverse transcription kit (Qiagen). cDNA generated by reverse transcription were used as template of qRT-PCR reactions next step. The reaction were performed using the SuperScript III Platinum SYBR green one-step qRT-PCR kit (Thermo Fisher). All the primers used here are presented in Table 1. GAPDH expression was used to normalize the relative mRNA expression levels: 2−^ΔCt^ (ΔCt = Ct[target gene]-Ct[GAPDH]). Roche LightCycle 480 platform were contributed in the qRT-PCR measurement and each reaction had performed with triplicates for statistical purpose.

**Table 1:** Primers used in RNA analysis.

Gene	Forward primer (5’-3’)	Reverse primer (5’-3’)
PLOD3	GACCCGGTCAACCCAGAGA	CTCCACCAACTGTTCGAGCC
FoxO3	TCACGCACCAATTCTAACGC	CACGGCTTGCTTACTGAAGG
Survivin	AGGACCACCGCATCTCTACAT	AAGTCTGGCTCGTTCTCAGTG
ACTB	CATGTACGTTGCTATCCAGGC	CTCCTTAATGTCACGCACGAT

### Western blot analysis

As soon as the cell culture complied with each experiment’s specifications, the lysis buffer for the RIPA assay was used to extract the total protein. The blot was then electrophoresed, incubated with 5% milk for two hours, and then incubated with primary antibody overnight at 4 °C.An enhancement chemiluminescence reagent (Thermo, USA) and a chemiluminescence imager (GE, USA) were then used to image the membrane after being incubated with the second antibody for 2 hours at room temperature.Antibodies used in present study were as follows: anti-PLOD3 (Abcam, ab89263, 1:1000); anti-cleaved Caspase 3 (CST, 9661 T, 1:1000); anti-PCNA (CST, 13110 T, 1:1000); anti-CCND1 (CST, 55506 T, 1:1000); anti-MMP9 (CST, 2270 S, 1:1000); anti-MMP3 (CST, 14351 S, 1:1000); anti-BCL2 (CST, 4223 T, 1:1000); anti-FOXO1 (CST, 2880 T, 1:1000); anti-FOXO3 (2497 S, 1:1000); anti-FOXO4 (CST, 2499 S, 1:1000); anti-FOXO6 (Proteintech, 19122- 1-AP, 1:500); anti-p21 (CST, 2847 T, 1:1000);anti-ubiquitination (CST, 58395 S, 1:1000); anti-Survivin (CST, 2808 T, 1:1000); anti-β-Actin (CST, 4970 T, 1:1000).

### Cell Viability Assay

MTT Cell Proliferation and Cytotoxicity Assay Kit (MTT) was chosen to detect the Cell viability. initially, cells were seeded in 96-well plates (NEST, China) in with 4000 cells per well in 100 μl volume of media, after 72 hours of incubation, 20 μL MTT were added each well to a final concentration of 5 μg/mL and reacted for 4 hours at 37 °C. Some enzymes within the mitochondria reduce MTT to form a dark lineal purple crystal product named formazan which can be completely dissolved in certain solvents. Therefore, after treating each well with 150 μL DMSO for 10 minutes, concentration of live cells can be measured according to the absorbance with a microplate reader at 450 nm wavelength.

### Colony Formation and Transwell Assays

Two cell lines were measured including Human GC SNU-216 (600 cells/well) and N87 (1000 cells/well) which were incubated in RPMI-1640 medium about10 days with 6-well plates. After culturing, the plates were gently washed with PBS and immediately added with 10% formalin to fix, then stained by 0.1% crystal violet. Following these steps, cells were counted well by well if there were no less than 50 cells each well after dried.

A transwell assay using or without Matrigel was conducted to assess cell invasion and migration potential [29]. In brief, after treatment, gastric cancer cells were collected and subsequently washed with PBS. To test the cell migration ability, noncoated membrane (24-well insert, pore size 8 μm; Corning) were used on upper chamber to culture 105 cells with DMEM containing 1% FBS. In the contrast, for invasion ability test, a Matrigel-coated membrane (dilution: 1:6) were applied in the upper chamber with the same number of cells and same medium formula. The lower chambers in both assays contained DMEM supplemented with 10% FBS, which served as chemoattractant. After incubated at 37 °C for 24 or 48, cells, which migrated or invaded to the lower surface of the membrane were fixed with 4% methanol. Then the membranes with cells were stained with crystal violet which make them easy to counted in 10 random 200× microscopic fields.

### Cell transfection

In order to knock down the expression of plod3, shRNA of PLOD3 sequence was cloned into a retroviral vector pMSCV. 293 T packaging cells served as retrovirus production. The pLKO.1- shRNA targeting the *plod3* oligonucleotide was sh1:5’-GGGAGGATATGATCATCATGT-3’; sh2: 5’- GGCCTTCTGTAAGAGCTTTCG-3’. We infected cells with this virus and a control virus at the same time and cured them by puromycin for 3 days before separating them to conduct further experiments.

### Microarray gene expression

The comparation of gene expression between tumor tissues from Trastuzumab -resistant and -sensitive PDX models were profiled by GeneChip® U133 Plus 2.0 arrays (Affymetrix). the GeneChip® Scanner 3000 (Cat#00- 00212, Affymetrix) were severed as the main detection machine and all results produced by Command Console Software 3.1 (Affymetrix) under default settings. Raw gene expression array data were analyzed by the algorithm Robust Multi-array Average, followed by log transformation and quartile normalization using the R package “simpleaffy”. The Spearman’s rank correlation coefficient was implemented to calculate similarity between two samples and a student’s t-test (*P* < 0.01) was applied to identify the association between gene expression and Trastuzumab sensitivity. Qiagen Ingenuity® Pathway AnalysisTM (IPA) software (www.qiagen.com/ingenuity) were purchased to help with the gene function analysis of differentially expressed genes. The complete dataset stored as a GEO (Gene Expression Omnibus) on the GEO database (www.ncbi.nih.gov/geo/; GEO accession number GSE90653).

### Tissue microarray (TMA) and immunohistochemistry

Embedded paraffin samples were stored at 4 °C after resection. In a previous article, we described how to construct the TMA and perform immunohistochemistry [30]. by the avidin-biotin-peroxidase complex method. We applied primary antibodies against the human-plod3 antigen to the slides after rehydration and microwave antigen retrieval, the slides were incubated at 4 °C overnight. Then, secondary antibody incubation was processed at 37 °C for 30 minutes. In this study, 3'3-diaminobenzidine tetra hydrochloride was used for staining and Mayer’s hematoxylin for counterstaining. In all assays, negative controls lacking the primary antibody were included.Three pathologists blinded to clinical characteristics assessed immunohistochemical staining, and discrepancies were resolved by consensus. The PLOD3 were stained by Anti-PLOD3 (Proteintech, 11027- 1-AP, 1:200) and then intensity was stratified as either high or low expression.

### In vivo animal assays

Chinese Science Academy (Shanghai, China) was the source of 6-week-old male nude mice used for setting up the mouse xenograft model in our study. We randomly implanted N87 cells in mice with lentiviruses (N87, N87-PLOD3^KD^; 3 × 10^6^) that had been approved by the ethics committee of Zhongshan Hospital of Fudan University. Every two weeks, tumor growth was measured, and the following equation was used to calculate growth: larger diameter × (small diameter)^2^/2. Approximately five weeks after the injection of gastric cancer cells, the mice were sacrificed, and their tumor tissues were surgically removed for hematoxylin and eosin staining.

### CHX chasing assays

A CHX chasing assay was used to determine the half-life of proteins. Briefly, indicated GC cells were incubated with CHX (100 μg/ml) for varying periods of time. In each group, total protein was extracted and immunoblotting was used to determine the levels of FoxO3.

### Ubiquitylation assay

The in vivo ubiquitylation level was determined by treating transfected cells with MG132 for 4 h prior to harvest and extraction in 1% SDS and 10 mM N- ethylmaleimide (Sigma). Then, extractions were denaturation at 95 °C for 10 min. Sonicated and diluted ten times in NP-40 lysis buffer, denatured protein extracts were immunoprecipitated using a total-β-Catenin antibody. The level of ubiquitination was then determined by immunoblotting with anti-Ub.

### Statistical analysis

Statistical analyses were processed by SPSS 20.0 software (IBM, Armonk, NY, USA). As standard, Experimental values were expressed as the mean ± standard error of the mean for continuous variables. Several types of tests were used to determine whether differences between the groups were significant, including Chi-squared tests, Student’s t tests, and Fisher’s exact probability tests. Nonparametric Mann-Whitney tests (Wilcoxon signed-rank tests) were applied when variances within groups were not homogeneous. Log-rank tests and Kaplan–Meier survival curves were used to analyze the relationship between PLOD3 expression and TTR or OS. *P* < 0.05 was considered statistically significant.

## Supplementary information

Original Data File

## Data Availability

The corresponding author will provide the original data used to support the findings of this study upon reasonable request.
